# Dependability of preservice clinical teaching scores using Generalizability Theory

**DOI:** 10.3389/fpsyg.2026.1728374

**Published:** 2026-03-03

**Authors:** Francis Ankomah, Ruth Keziah Annan-Brew, Kenneth Asamoah-Gyimah, Regina Mawusi Nugba, Eric Anane, George Oduro-Okyireh

**Affiliations:** 1 Department of Education and Psychology, University of Cape Coast, Cape Coast, Ghana; 2 Patton College of Education, Ohio University, Athens, OH, United States; 3 Institute of Education, University of Cape Coast, Cape Coast, Ghana; 4 Department of Interdisciplinary Studies, Akenten Appiah-Menka University of Skills Training and Entrepreneurial Development (AAMUSTED), Kumasi, Ghana

**Keywords:** dependability, generalizability theory, preservice teachers, reliability, teaching practice

## Abstract

**Introduction:**

The core of teacher training is to prepare preservice teachers to engage in teaching practices that enhance the acquisition of 21st-century skills. Clinical teaching is an integral part of teacher training. The processes involved in assessing preservice teachers’ clinical teaching experiences are not immune to error; therefore, when not well executed, they may compromise the validity and reliability of the scores derived.

**Methods:**

Our study applies Generalizability theory (G-theory) to examine sources of error in clinical teaching practice at a university in Ghana. We adopted a three-facet partially nested random balanced design, with occasion nested within rater, nested within person, and all crossed with items, denoted by (*o*: *r*: *p*) × *i*, to investigate clinical teaching observations involving 20 items, 107 raters, 2 occasions, and 208 preservice teachers.

**Results:**

We found that person-by-item interaction and the residual were the major sources of error. The generalizability and dependability coefficients were 0.91 and 0.90, respectively.

**Conclusion:**

We concluded that preservice teachers’ teaching competencies were assessed with high level of precision, though the assessment process was characterized by some errors. The study made useful recommendations to key stakeholders.

## Introduction

The National Council for Accreditation of Teacher Education in the United States (National Council for Accreditation of Teacher Education, 2010) posits that for teachers to be effective in 21st century classrooms, their education and training must focus on school-based experiences and deemphasize traditional academic preparation in which courses are taught in isolation, in favour of practical activities that occur in real-life settings. Teacher education programs must be rooted in clinical practice experience that is intertwined with content, pedagogy, and professional courses (Strieker et al., 2014). Globally, many nations and institutions evaluate the preparedness of preservice teachers through clinical teaching and experience. Hollins and Warner (2021) view clinical teaching as a component of the teacher preparation program that focuses on “the application of academic knowledge to practice in classrooms, schools, and communities where candidates learn to contextualize the curriculum, learning experiences, and other teaching practices for specific individuals and groups of students, p. 2.” This view of clinical teaching and experience provides a real-life simulation environment and an opportunity for preservice teachers to demonstrate their content, pedagogical knowledge, and skills. This provides the platform for bridging the gap between theory and practice. Clinical teaching has been referred to differently in the literature as teacher residencies, internships, practicum, student teaching (Hollins and Warner, 2021), and teaching practice in Ghana (Oppong, 2013; Agbalenyo et al., 2019).

The core of teacher training is to prepare preservice teachers to engage in teaching practices that foster lifelong learning among learners. The teaching practicum is an integral part of teacher education programs and of teacher training in general. It provides preservice teachers the opportunity to interact with students in classroom settings [Transforming Teacher Education and Learning (T-TEL), 2016]. One of the mandates of the Ministry of Education, Ghana (MoE-Ghana), is to enhance learning outcomes in Ghanaian schools through policy implementation measures (Ministry of Education, Ghana, 2017a). In executing this mandate, the Ministry emphasizes initial continuous teacher development aimed at improving the quality of the teaching force. The Ghanaian teacher education transformation agenda, as specified in the National Teacher Education Curriculum Framework (NTECF), centers on four pillars. The third and fourth pillars, which concern pedagogic knowledge and supported teaching in schools, require preservice teachers to focus on acquiring knowledge of pedagogy and assessment techniques. This is further strengthened by the National Teachers’ Standards for Ghana, a policy document that outlines the benchmark for professional practice for teachers in Ghana (Ministry of Education, Ghana, 2017b).

Consistent with cognitive apprenticeship theory, clinical teaching enables preservice teachers to learn the profession under the guidance of experienced, more knowledgeable teacher professionals (Collins et al., 1991). This apprenticeship requires that more knowledgeable professionals serve as mentors to preservice teachers and support their entry into the profession. Within the ambit of cognitive apprenticeship theory, learning is conceptualized as more cognitive than physical and is carried out through six processes: modeling, coaching, scaffolding, articulation, reflection, and exploration (Collins et al., 1989). During modeling, expert teachers demonstrate to preservice teachers. The mentors coach the preservice teachers by closely observing them and providing feedback. They also provide support to these preservice teachers and gradually withdraw this support to foster their mentees’ independence. The assumption is that, upon completing these, preservice teachers articulate, reflect on, and explore the new skills they have learned from clinical experience. The term preservice teacher is also referred to as student teacher (Hollins and Warner, 2021); hence, these concepts are used interchangeably in the literature.

Strieker et al. (2014) noted that performance-based assessment is an effective means of evaluating preservice teachers, particularly in an era where 21st-century skills are highly desired. Performance assessments can be meaningfully viewed as rater-mediated assessments because the ratings modeled in the psychometric analyses are obtained from judges (Engelhard, 2002). A central consideration in interpreting results from rater-mediated assessments is the quality of ratings (Johnson et al., 2009). Therefore, evidence of rating quality is a key component of the psychometric quality of rater-mediated assessments (American Educational Research Association, American Psychological Association, National Council on Measurement in Education, 2014).

Extant literature have shown that due to the stakes associated with teaching practice grades, some preservice teachers have expressed different opinions and perceptions, such as supervisors being biased and not knowledgeable in the area (Owusu and Brown, 2014; Kourieos, 2012), supervisors being quick in awarding grades, unfair ratings, overly supervised, consistently being given lower marks (Ngara and Magwa, 2017; Desai, 2012; Baidoo, 2016), inconsistencies among grades assigned by different supervisors (Oppong, 2013; Agbalenyo et al., 2019). Based on these perceptions, among others, it is appropriate to evaluate the entire teaching practice exercise. The teaching practice assessment procedure, as a measure of human behavior, is not immune to errors. This makes the scores obtained susceptible to human error and bias, thereby reducing their validity and reliability.

Several studies have empirically examined the errors in the scores generated through rater-mediated teaching practice observation assessments with efforts of taking pragmatic measures to reduce these errors using approaches such as many-facet Rash model (Wind et al., 2018; Wind, 2019; Jones and Bergin, 2019), G-theory (Briggs and Alzen, 2019; Casabianca et al., 2015; Weston et al., 2021; Jones et al., 2022; Reinholz et al., 2021; Oduro-Okyireh, 2021; Huijgen et al., 2017; White, 2017), traditional factor analysis (Rogers, 2016), latent class analysis (Halpin and Kieffer, 2015), combination of traditional factor analysis and item response theory (IRT) (Molina et al., 2020), traditional factor analysis and bifactor model (Stern, 2016). These studies revealed that items, raters, occasion, and other confounding factors (residuals) beyond the scope of the studies influenced the scores in their measurement. Notably, the majority of the previous studies focused on observing in-service teachers (Wind, 2019; Jones and Bergin, 2019; Briggs and Alzen, 2019; Casabianca et al., 2015; Jones et al., 2022; Jones and Bergin, 2019; Chen and Terada, 2021) with only a few focusing on preservice teachers (Weston et al., 2021; Oduro-Okyireh, 2021; Stern, 2016). Clearly, these are distinct situations with respect to teaching practice observations. Therefore, the applicability of previous studies’ findings to preservice teachers, as in the current study, is compromised for several reasons. First, different targets of measurement are addressed in both situations (i.e., pre- and in-service teachers); hence, they are not exchangeable. In-service teachers are certified teachers with many years of experience; by definition, they approach teaching differently from pre-service teachers who are not yet certified. Second, the goals and expectations differ between the two situations. Whereas the focus is to grade and certify preservice teachers, the focus of in-service is to determine teaching effectiveness for either promotion or other formative use. This implies that varied exemplars and descriptors are likely to be used for each situation.

Another point noteworthy is that only a single similar study appears to have been conducted in Ghana (Oduro-Okyireh, 2021). There is predominance of previous studies in the US (Wind, 2019; Weston et al., 2021; Jones et al., 2022; Jones and Bergin, 2019; Niu, 2021), the Netherlands (Huijgen et al., 2017), and Pakistan (Molina et al., 2020). The Ghanaian study by Oduro-Okyireh (2021), using G-theory, reported a very high level of dependability in students’ teaching practice internships at a university different from that in the current study. What was prominent about Oduro–Okyireh’s study was that it considered only occasion as the facet out of other possible sources of error, such as rater and item that surrounded the measurement procedure. Not considering many facets limits the universe of admissible observations and generalizations. Additionally, more errors remain unaccounted for, reducing the validity and reliability of the generated scores. It is important to state that the validity and reliability of the teaching practice observation information largely rely on the knowledge and accuracy of the supervisors who double as raters. On this backdrop, an investigation into the potential sources of errors in the teaching practice observation scores with the application of G-theory would provide an empirical basis for measures to improve the practice and the direction of training for supervisors. This study, therefore, applies G-theory to:

1. examine the sources of errors associated with the teaching practice observation;
2. determine the reliability (G-coefficient and index of dependability) of the teaching practice observation;
3. optimize the number of raters, occasions, and items required to obtain an optimum level of dependability.

### Study context

The teaching practice exercise is primarily conducted in two segments: on- and off-campus. On-campus is an internal practicum session in which preservice teachers teach their peers. For the off-campus, preservice teachers move outside to teach students in real classroom settings. The essence of this teaching practice activity is to equip preservice teachers with professional skills, competencies, and a positive attitude toward the teaching profession [Transforming Teacher Education and Learning (T-TEL), 2016]. Supervisors observe and rate the preservice teachers as they deliver their lessons in the classroom (Owusu and Brown, 2014). Scores from these observations are used to grade preservice teachers and contribute to their final cumulative grade point average (CGPA). In the university under investigation, preservice teachers engage in both on- and off-campus teaching practices. The former constitutes 3 credit hours, while the latter constitutes 12 credit hours. Off-campus teaching practice, specifically, accounts for approximately 80% of the total credit hours for that semester. This suggests that grades obtained by preservice teachers in off-campus teaching practice are critical to their GPAs and CGPAs. A 20-item observation protocol is used for the teaching practice exercise. The teaching practice assessment exercise at the university under investigation is a rater-mediated performance assessment. Therefore, it is pertinent to ensure the quality ratings of preservice teachers assigned to teaching practice supervisors. When teaching practice supervisors under- or over-rate preservice teachers, they introduce errors into students’ scores, which greatly affect the validity and reliability of the grades obtained and, by extension, the final CGPAs.

### Theoretical framework

This study was conducted using Generalizability theory as the lens of investigation. Generalizability theory (G-theory) is a body of statistical theories concerned with the reliability of measurement in the behavioral sciences (Shavelson and Webb, 1991). With G-theory, in a single analysis, multiple sources of variability (measurement errors) can be estimated simultaneously. Brennan (2001) indicated that the primary purpose of G-theory is to estimate variance components that are linked with a particular universe of admissible observations. The universe of admissible observations comprises all possible observations on an object of measurement that a decision maker considers to be acceptable substitutes for the observation in hand. Put differently, the universe of admissible observations is the union of the various conditions of measurement of the facets involved in a particular study. Generalizability theory estimates the amount of error attributable to each factor or condition under which a particular measurement is carried out (Tindal et al., 2010). Brennan (2001) classified facets as facets of differentiation and facets of instrumentation. The facets of differentiation are the object of measurement, whereas the facets of instrumentation, which are also called the facets of generalization, comprise other sources of variance than the object of measurement.

Cardinet et al. (2010) noted that the facets of instrumentation can be defined as “fixed,” “random,” or “finite random” based on their sampling status. A facet is “fixed” when all levels are featured in the data set (i.e., no sampling of levels has occurred). A facet is “random” when the levels included in the analyses are randomly selected from the respective population or universe. A “finite random” facet is also referred to as “mixed” because, within a finite universe, a random sampling can be conducted. According to Brennan (2001), a design can be “complete” and “balanced” when all possible interactions have been considered (i.e., complete) and all facets in the study have the same number of elements (i.e., balanced). Such designs have equal amounts for all facets; for example, all raters have the same number of occasions, and all students were observed on the same number of occasions. The use of a complete and balanced design helps minimize overall error variance since there is no missing data that might inflate the error variance. However, the use of such designs is characterized by a reduction in the size of the dataset (Brennan, 2001). An unbalanced design has an unequal number within facets; for example, every student has a different number of supervisions, and every occasion has a different number of supervisors.

Various designs are employed in G-theory. The design could either be nested or crossed. Two facets are crossed when any levels of one facet can interact with any levels of the other facet (e.g., preservice teachers are crossed with observation items when all preservice teachers are assessed using all observation items). One facet is nested within another facet when the nested facet has different levels for certain facets—it is not crossed; this happens in all unbalanced designs. For instance, in a study, if all preservice teachers (p) are observed with the same items (i), then preservice teachers are crossed with items. This design is denoted as *p x i*. However, if each preservice teacher is observed with a different set of items, the data are structured as items nested within preservice teachers, denoted *i:p*. G-theory conducts its analysis in two steps. First, a generalizability study or G-study is conducted to (a) calculate variance components for multiple sources of variance (i.e., facets) and (b) estimate the reliability coefficients in the form of generalizability and dependability coefficients (Cronbach et al., 1972). A “decision study or D-study makes use of the information provided by the G-study to design the best possible application of the social science measurement for a particular purpose” (Shavelson and Webb, 1991, p. 12). In the planning of a D-study, the researcher defines a universe of generalization, the set of facets and their levels to which he or she wants to generalize, and specifies the proposed interpretation of the measurement. D-studies help researchers or educators make decisions regarding efficient use of logistics and resources to yield optimum results. In sum, while G study identifies the various sources of error variance, D study, in contrast, provides clarity on the conditions under which error variances can be minimized to yield the best desired result. Regarding the G-coefficient, there is currently no consensus on an acceptable value. While some scholars recommend a minimum of 0.65 (Ho and Kane, 2013), others recommend 0.80 as an appropriate precision measurement (Shavelson and Webb, 1991; Cardinet et al., 2010). Brennan and Kane (1977) also indicate that the *Φ* should be ≥0.7 for research purposes, ≥0.8 for formative evaluations, and ≥0.9 for summative evaluations.

### Using G-theory in the context of this study

In the current study, items, occasions, and raters are the facets; therefore, the universe of admissible observations comprises all possible raters, items, and occasions that we are willing to consider as having potential effect on preservice teachers’ ability in teaching during the off-campus. The universe of admissible observation is the totality of facets considered in the study (Shavelson and Webb, 1991). Universe of generalization, in contrast, is the group of facets, as well as their levels, to which one wants to generalize. The theory makes estimates on how dependable generalizations or inferences are made from a preservice teacher’s observed score (teaching ability) to the score they would get in all possible repeated teaching practice evaluations (universe score).

Within the context of the current study, the measurement process involved in the off-campus teaching practice exercise is seen to have been affected by errors; therefore, the observed scores derived for the preservice teachers have errors embedded in them. The current study applies G-theory to the measurement of teaching practice to dissociate errors from the true abilities of preservice teachers (Brennan, 2001). In line with G-theory, this study proposes that errors can be portioned and quantified to determine their relative contributions. On this premise, this study identifies one of the errors as emanating from the items. In this regard, issues related to item ambiguity, inappropriate difficulty levels, and unclear response options, among others, affect the precision of the data obtained. Further, inconsistencies among raters in awarding scores for the teaching practice exercise may contribute to errors in the off-campus teaching practice measurement. Additionally, inconsistency among raters in rating students from one occasion to the next on the same items may also contribute to some error.

Moreover, the current study seeks to assess potential errors arising from interactions among facets. Within the framework of G-theory, sources of error were modeled as variance components, each with a corresponding contribution, which was expressed as a percentage for ease of comparison. Knowledge of the possible sources of variances, as well as their magnitudes, helped suggest measures to reduce these errors in off-campus teaching observations. In addition to these variances, the relative and absolute error variances were estimated. These comprise a combination of the various sources of variability. The relative and absolute error variances quantified the magnitude of error in normative and criterion interpretation of the observation scores from teaching practice (Shavelson and Webb, 1991; Brennan, 2001). Conversely, the standard error of measurement is estimated. Finally, the G-coefficient and index of dependability are computed from the relative and absolute error variances. These indices gave evidence of the reliability of the observation data. More importantly, G-theory was used to model the numbers of supervisors, items, and occasions, which, when combined, would yield optimal reliability of the teaching practice observation data.

## Materials and methods

### Design

**Figure 1:**
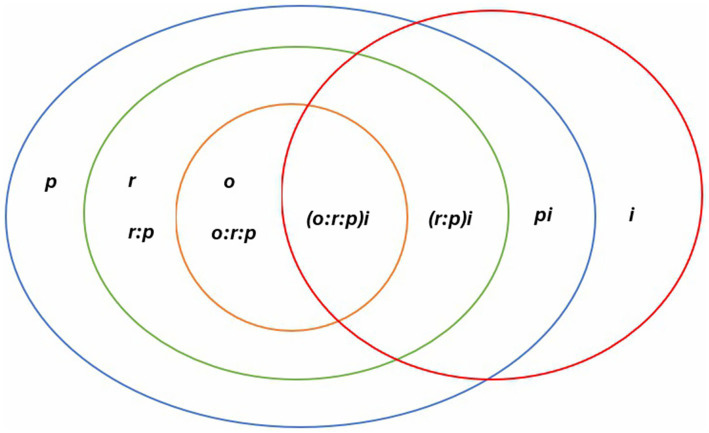
Variance partition for the teaching practice observation.

This study was ingrained in the positivist paradigm. Ontologically, this study rests on the belief in a single, definite, observable, and measurable reality (Creswell and Plano Clark, 2018). Specifically, the three-facet partially nested random balanced design, with occasion nested within rater, nested within person, and all crossed with items denoted by (*o*: *r*: *p*) × *i*, was employed (Shavelson and Webb, 1991; Shavelson and Webb, 2006). The *o*, *r*, *p*, and *i* represent occasion, rater, preservice teacher, and item, respectively. Occasion, rater, and item were considered as the facets in this study. The preservice teacher (p) was the object of measurement. The design used in this study implied that each preservice teacher was rated by a different set of raters and that every rater rated on different occasions; however, on every occasion, every rater, every preservice teacher, all items were used. From the design, seven possible sources of error variances were estimated from the off-campus teaching practice observation (see Figure 1). The choice of the design aligns with how the clinical teaching exercise was conducted. The clinical teaching was conducted during the first semester of the final year for the preservice teachers. The entire exercise took place in 4 months. During this period, there were no specific or specified days (occasions) for the observation. This means that every rater determines when (occasion) they want to observe the preservice teacher. For instance, while one rater may decide to observe the preservice teacher in the first week, another rater may choose to observe in the third or fourth week. The ratings were conducted on different occasions. The minimum number of supervisions a preservice teacher was supposed to have was two; however, there was no maximum. While some preservice teachers had two ratings, others had up to 14 ratings by different combinations of raters. The observation protocol had 20 items. Every rater used all 20 items for every preservice teacher, and for every occasion, the same 20 items were used.

### Study data

The data used in this study were secondary data. Using the census method, a total of 4,379 observation cases of 1,170 preservice teachers, conducted by 304 supervisors during off-campus teaching practice observation for undergraduate regular students at a university in Ghana in the 2020–2021 academic year, were used in this study. Different streams of undergraduate programs are offered in the university under investigation. These include regular, sandwich (this happens during school vacation periods), and distance learning (weekends). The following exclusion criteria were adhered to. First, preservice teachers with only one supervisor were excluded. Observation cases where all items on the observation protocol were not checked were also not considered. Further, supervision for practical lessons was not considered in this study. This resulted in 20 items, 146 raters, and 781 preservice teachers. The occasions ranged from 2 to 14. In total, 87,555 rows and 5 columns of cases were involved when the data were transformed into the wide format for use in the analysis. This resulted in 437,775 individual cells, which was relatively large and resulted in multiple running issues. However, due to model complexities involved in the multiple nested conditions and the difficulty in getting a supercomputer to run the analysis, two occasions were randomly selected. To ensure that the two randomly selected occasions were appropriate for the analysis, three other sets of two randomly selected occasions were generated, and when the results for these four datasets were compared, there were no substantial differences. This procedure of randomly selecting levels of a facet was implemented in accordance with the recommendation of Shavelson and Webb (1991). Shavelson and Webb (1991) noted that “randomly deleting levels of a facet to create a balanced design has little effect on the variance components and allows a wide variety of computer programs to be used” p. 73. Shavelson and Webb (1991) further recommend the comparison of the “results of several random deletions to make sure that any particular selection is not unusual” p. 73. Having gone through all these processes, the final version of the data had 208 preservice teachers, 107 raters, and 2 occasions.

The observation protocol used by the university for the clinical teaching practice had 20 items, which comprised four dimensions: Lesson plan (5 items), Teaching methodology and delivery (11 items), Classroom organization and management (2 items), and Professional commitment (2 items). The items had the following ratings: “5” very good, “4” good, “3” satisfactory, “2” unsatisfactory, “0/1” poor. Three research assistants were trained on basic ethical principles in research, and they assisted in the data collection. Ethical clearance was obtained from the Institutional Review Board of the University of Cape Coast, Ghana, with reference number: I0RG0011497. Ethical issues such as confidentiality, anonymity, and data protection were adhered to.

### Data analysis

The G-theory analysis was conducted using the R program and the EduG software. The “aov” function in the R program was used to estimate the sum of squares and degrees of freedom, and these were subsequently transferred to the EduG software for the estimation of the variance components and the generalizability coefficients. Based on the design, seven variance components are estimated:

Variance components:


$$\sigma^{2}(\text{Xproi})=\sigma_{p}^{2}+\sigma_{r:p}^{2}+\sigma_{o:r:p}^{2}+\sigma_{(r:p)i}^{2}+\sigma_{\mathrm{pi}}^{2}+\sigma_{i}^{2}+\sigma_{(o:r:p)i}^{2.}$$

The first variance component is the variance for persons ( $\sigma_{p}^{2}$ ). This is also referred to as the universe score. The second variance component is raters nested persons ( $\sigma_{r:p}^{2})$ . This component represents the variation for the different raters rating the preservice teachers on their teaching practice. The third variance component is occasions nested in raters nested in persons ( $\sigma_{o:r:p}^{2}$ ). This is largely the variation emanating from the different sets of raters for different preservice teachers on different occasions. The fourth source of variability is raters nested in persons crossed with items ( $\sigma_{(r:p)i}^{2}$ ). This component of error represents the variability resulting from the combination of rater and person over item. This implies that each preservice teacher was rated by a different supervisor, but the raters used the same items to rate all the preservice teachers. The fifth component of variance is persons crossed with items ( $\sigma_{\mathrm{pi}}^{2}$ ), implying the systematic variability emanating from the preservice teachers from one item to another. The sixth component of variance is items ( $\sigma_{i}^{2}$ ). This variance proportion represents the variability in the difficulty of the test items. The last variance component is the residual ( $\sigma_{(o:r:p)i}^{2}$ ).

### Error classification

In this study, the errors estimated were classified as relative error and absolute error. Based on the estimations of the variance components already identified in the study, the relative and absolute errors are shown as follows:


$$\text{Relative error}=\sigma_{\mathrm{Rel}}^{2}=\frac{\sigma_{r:p}^{2}}{n_{r}}+\frac{\sigma_{o:r:p}^{2}}{n_{o}n_{r}}+\frac{\sigma_{\mathrm{pi}}^{2}}{n_{i}}+\frac{\sigma_{(r:p)i}^{2}}{n_{r}n_{i}}+\frac{\sigma_{(o:r:p)i}^{2}}{n_{o}n_{r}n_{i}}$$

The relative error is the sum of all the various variance components that are associated with the preservice teachers (object of measurement). The relative errors are used in normative interpretations of preservice teachers’ abilities as well as relative comparison of severity of raters, and so forth. The relative error model does not include the main effects, which in this case were items (Shavelson and Webb, 2006). This is because the same items were used on all the preservice teachers; therefore, any variation in the items applies to all the preservice teachers, hence their normative positions are not affected. Put differently, any error from the item affects all the preservice teachers in the same way. The model for absolute error with its components is expressed as follows.


$$\begin{array}{l} \text{Absolute error}=\sigma_{\mathrm{Abs}}^{2}=\frac{\sigma_{i}^{2}}{n_{i}}+\frac{\sigma_{r:p}^{2}}{n_{r}}+\frac{\sigma_{o:r:p}^{2}}{n_{o}n_{r}}+\\ \frac{\sigma_{\mathrm{pi}}^{2}}{n_{i}}+\frac{\sigma_{(r:p)i}^{2}}{n_{r}n_{i}}+\frac{\sigma_{(o:r:p)i}^{2}}{n_{o}n_{r}n_{i}} \end{array}$$

The absolute error comprises the combination of all variance components apart from the variance from the preservice teacher facet, which is the object of measurement in this study. The absolute error is used for absolute decisions, such as criterion-referenced interpretations. This error includes the components of relative error as well as all the main effects, which in this case was item. The inclusion of the main effect is on the assumption that, though any variation in item affects all preservice teachers similarly, the difficulty or otherwise of any of the items duly affects the estimation of preservice teachers’ teaching ability; therefore, it is included in the error estimation (Shavelson and Webb, 2006). Relatively, the absolute error is always greater than the relative error. The square root of both the relative and absolute error variances gives the standard error of measurement for each of them.

### Generalizability and dependability coefficients

Information obtained from the relative and absolute error variances was used in the computation of the G- and phi-coefficients. The G-coefficient is presented as follows:

$\text{Generalizability coefficient}\;(E\rho_{\mathrm{Rel}}^{2}$ ) = $\frac{\sigma_{p}^{2}}{\sigma_{p}^{2}+\sigma_{\mathrm{Rel}}^{2}}$

The generalizability coefficient is the proportion of the variance of the universe score to the sum of the variance of the universe score and the variance of the relative error. The universe score variance is the true score, whereas the sum of the variance of the universe score and the variance of the relative error is the observed score. Therefore, the G-coefficient can be likened to the reliability estimated in the classical measurement approach. The G-coefficient ranges from 0 to 1, where higher values indicate higher reliability. Since the G-coefficient has a component of relative error, the G-coefficient is used for relative decisions.

The dependability coefficient is the proportion of the variance of the universe score to the sum of the variance of the universe score and the variance of the absolute error. It is expressed as follows:


$$\text{Dependability coefficient}\;(\phi )=\frac{\sigma_{p}^{2}}{\sigma_{p}^{2}+\sigma_{\mathrm{Abs}}^{2}}$$

Like the G-coefficient, the dependability coefficient rather has the component of absolute error; therefore, it is used for criterion-referenced interpretations. The dependability coefficient also ranges from 0 to 1. The generalizability coefficient is higher than the dependability coefficient. In terms of interpretations of the generalizability and dependability coefficients, scholars recommend 0.80 as an appropriate precision measurement (Shavelson and Webb, 1991; Cardinet et al., 2010). Alternatively, Brennan and Kane (1977) also recommend that the *Φ* should be ≥0.7 for research purposes, ≥0.8 for formative evaluations, and ≥0.9 for summative evaluations.

## Results

The mean scores for the items ranged from 4.08 to 3.58 (out of 5.0), with standard deviations of approximately 0.5. The items were not highly correlated with one another (Table 1).

**Table 1:** Descriptive statistics and correlations among items.

	IT1	IT2	IT3	IT4	IT5	IT6	IT7	IT8	IT9	IT10	IT11	IT12	IT13	IT14	IT15	IT16	IT17	IT18	IT19
I2	0.306^**^																		
IT3	0.300^**^	0.317^**^																	
IT4	0.148^**^	0.226^**^	0.313^**^																
IT5	0.227^**^	0.318^**^	0.337^**^	0.270^**^															
IT6	0.365^**^	0.250^**^	0.244^**^	0.198^**^	0.149^**^														
IT7	0.113^**^	0.193^**^	0.250^**^	0.169^**^	0.287^**^	0.150^**^													
IT8	0.197^**^	0.141^**^	0.153^**^	0.165^**^	0.206^**^	0.165^**^	0.193^**^												
IT9	0.145^**^	0.185^**^	0.167^**^	0.126^**^	0.176^**^	0.163^**^	0.218^**^	0.187^**^											
IT10	0.100^**^	0.204^**^	0.138^**^	0.194^**^	0.203^**^	0.144^**^	0.193^**^	0.172^**^	0.205^**^										
IT11	0.134^**^	0.154^**^	0.190^**^	0.482^**^	0.146^**^	0.142^**^	0.206^**^	0.185^**^	0.212^**^	0.185^**^									
IT12	0.161^**^	0.184^**^	0.196^**^	0.101^**^	0.210^**^	0.128^**^	0.226^**^	0.181^**^	0.172^**^	0.123^**^	0.121^**^								
IT13	0.145^**^	0.237^**^	0.171^**^	0.153^**^	0.209^**^	0.136^**^	0.332^**^	0.173^**^	0.313^**^	0.158^**^	0.228^**^	0.252^**^							
IT14	0.229^**^	0.212^**^	0.228^**^	0.140^**^	0.344^**^	0.230^**^	0.295^**^	0.159^**^	0.159^**^	0.179^**^	0.110^**^	0.311^**^	0.271^**^						
IT15	0.108^**^	0.264^**^	0.138^**^	0.188^**^	0.161^**^	0.105^**^	0.253^**^	0.183^**^	0.282^**^	0.159^**^	0.210^**^	0.164^**^	0.265^**^	0.188^**^					
IT16	0.127^**^	0.192^**^	0.134^**^	0.137^**^	0.107^**^	0.198^**^	0.139^**^	0.156^**^	0.143^**^	0.129^**^	0.201^**^	0.094^**^	0.154^**^	0.134^**^	0.366^**^				
IT17	0.150^**^	0.217^**^	0.135^**^	0.160^**^	0.147^**^	0.123^**^	0.217^**^	0.191^**^	0.244^**^	0.205^**^	0.157^**^	0.189^**^	0.282^**^	0.177^**^	0.239^**^	0.147^**^			
IT18	0.173^**^	0.215^**^	0.140^**^	0.105^**^	0.191^**^	0.158^**^	0.195^**^	0.207^**^	0.244^**^	0.119^**^	0.119^**^	0.194^**^	0.257^**^	0.171^**^	0.256^**^	0.185^**^	0.394^**^		
IT19	0.204^**^	0.220^**^	0.239^**^	0.190^**^	0.300^**^	0.194^**^	0.225^**^	0.159^**^	0.162^**^	0.223^**^	0.124^**^	0.210^**^	0.187^**^	0.332^**^	0.184^**^	0.136^**^	0.196^**^	0.241^**^	
IT20	0.168^**^	0.201^**^	0.118^**^	0.113^**^	0.154^**^	0.160^**^	0.143^**^	0.143^**^	0.121^**^	0.120^**^	0.159^**^	0.110^**^	0.142^**^	0.193^**^	0.236^**^	0.302^**^	0.160^**^	0.204^**^	0.229^**^
Mean	4.08	3.85	3.87	3.71	3.98	3.97	3.83	3.88	3.71	3.83	3.71	3.86	3.89	4.03	3.58	3.62	3.86	3.82	3.91
SD	0.56	0.51	0.51	0.66	0.49	0.62	0.52	0.52	0.56	0.56	0.65	0.50	0.55	0.50	0.58	0.61	0.47	0.49	0.61

**Correlation is significant at the 0.01 level (two-tailed); IT, item.

### Sources of errors associated with the teaching practice observation

This study identified the sources of variability in the data obtained from the off-campus teaching practice observation (see Table 2). The residual term accounted for the largest proportion of variability in the teaching practice observation data (41.5%). This signifies the magnitude of error arising from the confounding effects of occasion and rater combinations across the different preservice teachers observed with the same items, as well as other systematic and unsystematic errors that were not considered in this study. This was followed by the person-by-item interaction (33.7%). This implies that the preservice teachers systematically differed in the ratings they received across items, averaged over raters and occasions. There was a substantial difference among preservice teachers in their teaching abilities (17%). This result may partly be attributable to prior teaching experience among preservice teachers. The item was the fourth source of variability (3.1%). Rater nested within person; all crossed with item were negligible. Person-by-item interaction contributed over 96 and 88% of relative and absolute error variances. Similar results were found across all dimensions. In all, the residual and person-by-item interaction were the major sources of error.

**Table 2:** Sources of variance for overall inventory.

Source	df	SS	MS	Variance ( $\sigma^{2}$ )	
				Component	%
Person (*p*)	207	661.8	3.197	0.00104	17.0
Rater: person (*rape*)	14,352	143.5	0.010	0.00012	2.0
Occasion:rater:person (*o:r:p*)	14,560	84.7	0.006	0.00016	2.7
Item (*i*)	19	108.8	5.726	0.00019	3.1
Person × item (*pi*)	3,933	1136.9	0.289	0.00205	33.7
(Rater: person) by item (*r:p*) × *i*	272,688	519.2	0.002	−0.00031	0.0
(Occasion:rater:person) by item (*o:r:p*) × *i,e*	276,640	699.3	0.003	0.00253	41.5
Total		3,354.2			100.0

SS, sum of squares; df, degrees of freedom; MS, mean square.

### Reliability (G-coefficient and index of dependability) of the teaching practice observation

We ascertained the reliability estimates for both absolute and relative interpretations. Across all four dimensions of the teaching practice observation protocol, professional commitment had the least reliable estimates for both relative and absolute, and these were equal (E $\rho_{\mathrm{Rel}}^{2}$ = Φ = 0.51, see Table 3). The teaching methodology dimension had the highest reliability estimates for both relative (0.82) and absolute (0.80). Overall, the reliability estimates for the entire teaching practice observation protocol were 0.91 for relative and 0.90 for absolute interpretations. In sum, reliability estimates for the entire inventory and all dimensions, except the professional commitment dimension, exceeded 0.70.

**Table 3:** Generalizability and dependability coefficients.

Scale/dimension	Coefficients	
	E $\rho_{\mathrm{Rel}}^{2}$	Φ
Lesson plan	0.78	0.76
Teaching methodology	0.82	0.80
Classroom organization	0.74	0.74
Professional commitment	0.51	0.51
Overall inventory	0.91	0.90

### Optimization of raters, occasions, and items required to obtain an optimum level of dependability

**Figure 2:**
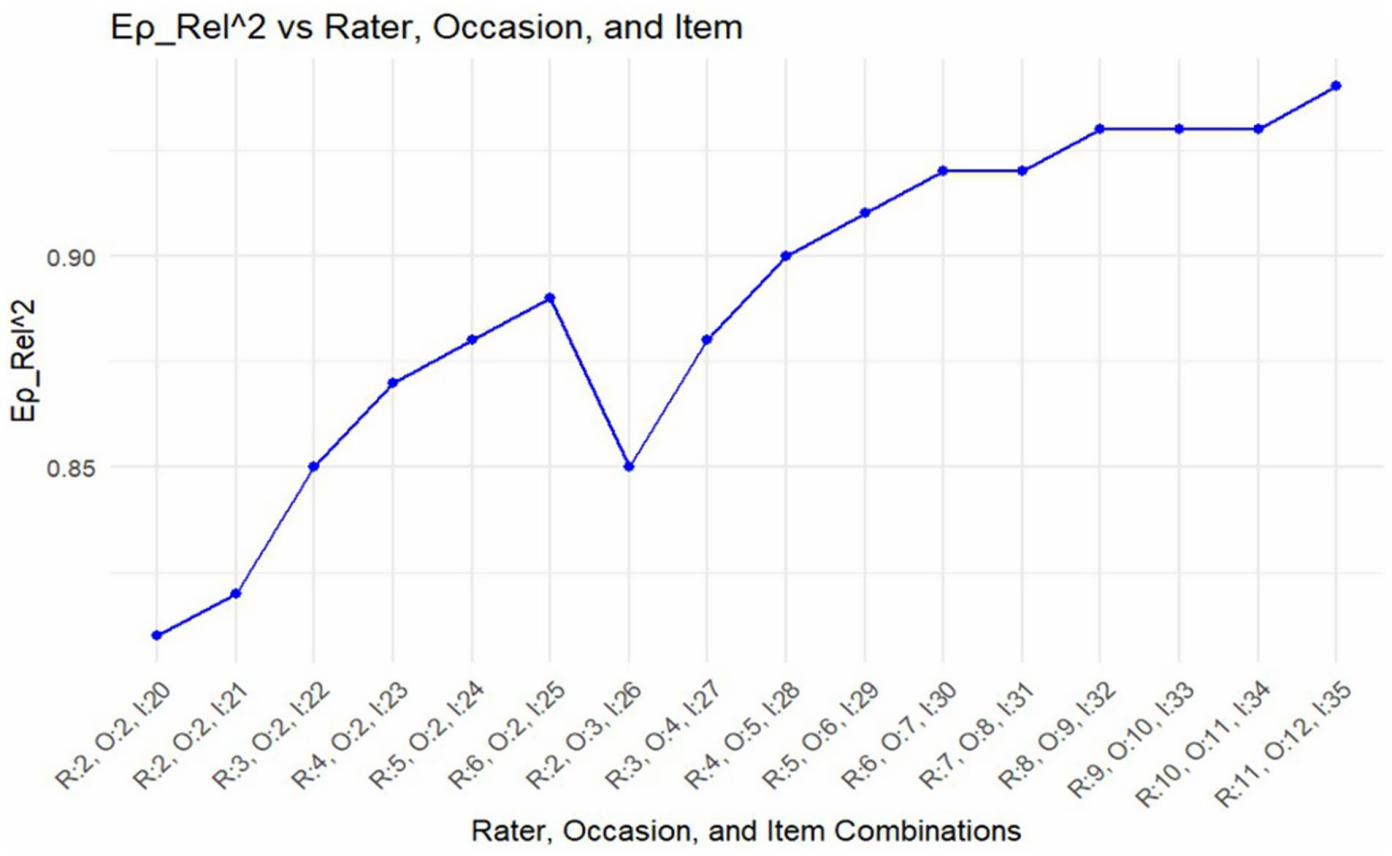
Optimization of occasion, rater, and item combination and their corresponding G-coefficients.

**Figure 3:**
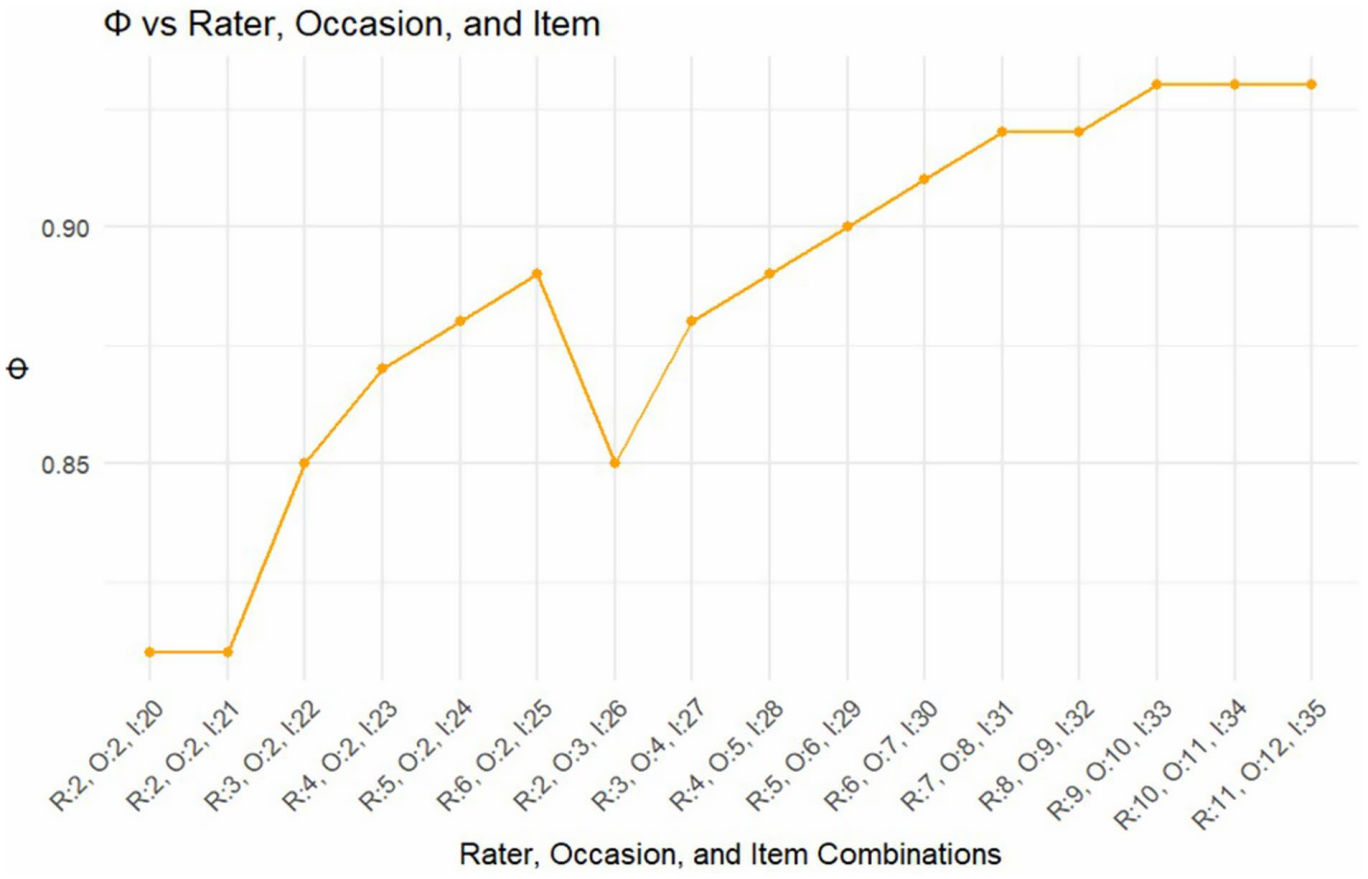
Optimization of occasion, rater, and item combination and their corresponding Phi-coefficients.

Finally, we empirically made projections on the number of: (a) raters, (b) supervisions, and (c) items necessary to yield an optimum level of dependability. Several simulations were performed by increasing and/or decreasing the number of elements within the facets. The results showed that, holding the number of occasions constant at two, both the g- and phi-coefficients increased from 0.81 to 0.89 when the number of raters was increased from two to six, and concurrently increasing the number of items from 20 to 25. Further, both g- and phi-coefficients increased to 0.94 and 0.93, respectively, when the number of raters increased from four to 11, while occasions also increased from six to 12, and items increased from 29 to 35 (see Figures 2, 3).

## Discussion

In this study, we sought to identify the sources of error in measuring preservice teachers’ competency. From the entire teaching practice procedure, possible factors/facets such as rater, occasion, and item were considered. We found that the residual contributed the majority of the source of error, corroborating with several other studies (Oduro-Okyireh, 2021; Semmelroth, 2013). This implies that quite a substantial amount of variation is due to the confounding effects of the occasion, rater, and person combination, all crossed with item as well as other sources of error beyond the scope of this study. This result could be reasonably explained by the combination of occasion, rater, and person. It must, however, be noted that the study by Oduro-Okyireh (2021) considered only occasion as a facet. As Shavelson and Webb (1991) noted, the inclusion of multiple facets in a study helps identify additional sources of variability and thereby reduces unexplained variability. In the teaching practice exercise examined in this current study, the preservice teachers were supervised by different raters on different occasions. Although it was not clearly established in this current study, it is possible that the preservice teachers were given higher scores on one occasion and a lower score on another occasion, either by the same or different raters. The nested design makes it difficult to draw a definite conclusion. In another related study, Jones et al. (2022) found that the residual accounted for moderate variation (22%), though it was not the largest in their study. Overall, our findings align with perceptions held by preservice teachers in previous studies (Oppong, 2013; Agbalenyo et al., 2019; Owusu and Brown, 2014; Desai, 2012; Baidoo, 2016). The studies reported inconsistent grades across different supervisors, consistently lower scores, and unfair ratings, among other issues.

Furthermore, our study revealed that person-by-item interaction contributed the second-highest variability, accounting for 33.7% of the overall variability and 21.3–34.5% across all four dimensions of the teaching practice observation protocol. Additionally, it contributed more than 80% in terms of relative and absolute error variances. This means that the preservice teachers systematically differed in their relative standing in teaching competence across items. Thus, there was a substantial difference in the ratings they obtained on the various items when averaged over raters and occasions. Thus, a preservice teacher who performs well on Item 2, for instance, may not necessarily perform well on all items. This finding is in harmony with Weston et al. (2021), who found that teacher observation by item was the highest source of error in their study. This result may suggest inconsistency in the scores from one item to another, either by the same supervisor or different supervisors. This aligns with a view shared by many preservice teachers in previous studies (Oppong, 2013; Agbalenyo et al., 2019). This may be attributed to the lack of agreement among supervisors.

From a different perspective, we argue that the items may vary in difficulty. In this case, the use of the same items for all students may be quite problematic because our results have shown that the preservice teachers differ systematically in their teaching competency. Hence, the tendency is that preservice teachers with the same abilities obtained different scores on a specific item. Alternatively, certain items may disadvantage some preservice teachers while favoring others. For example, an item focusing on teaching methodology and delivery may disadvantage preservice teachers with less experience. This situation may not be helpful for relative comparisons among preservice teachers, and for absolute thresholds, where preservice teachers are expected to achieve at least 50% (i.e., grade D) to be considered pass. Although this result was counterintuitive, it calls for a reconsideration of how preservice teachers should be assessed. By implication, the preservice teachers are unique in their own ways and have diverse needs. Subjecting all preservice teachers to the same observation items may imply that they are being treated equally, without regard for their unique differences. This may not be accommodating enough to cater for their diverse needs.

Findings from the current study showed that across all dimensions and overall, the preservice teachers systematically differed in their teaching competencies. It is important to note that the observation items may not have the same difficulty levels as those used in this study, given the inherent variability in the preservice teachers’ competency levels. Therefore, in line with contemporary measurement practices, one would expect that the observation items be matched with the competencies of preservice teachers in a more adaptive way, such that every preservice teacher’s ability would be equitably measured. It was further revealed that the rater nested within person, crossed with items, accounted for the least variability and contributed negligible amounts (0%) across all items. This implies that the different supervisors’ ratings of different preservice teachers with the same observation protocol did not necessarily contribute to error. The same can be said of a rater nested within person. Similarly, items contributed relatively small but non-negligible amounts of error across all dimensions, as did the overall inventory. This error could be attributed to factors such as item clarity, number of items, and item sampling, among others. Error variance attributable to the rater could not be estimated using G-theory because the rater facet was nested within persons. However, the confounding effects of the rater facet with other facets were not large, but they were not negligible, agreeing with a couple of past studies (Briggs and Alzen, 2019; Semmelroth, 2013). Consistent with this study, Briggs and Alzen (2019) found that the rater facet was the least variable and the occasion the largest source of variability in teacher observation scores. On the contrary, Casabianca et al. (2015) and White (2017) found that the rater was the largest source of variability. A plausible explanation for the contrast is differences in the objects being measured. Casabianca et al’s. (2015) study involved in-service teachers and thus describes a different measurement context.

Our study further assessed the reliability of the scores generated from the teaching practice exercise. Within the framework of G-theory, reliability coefficients of 0.91 and 0.90 were found for the absolute and relative interpretations, respectively, for the overall teaching practice observation protocol. Except for the professional commitment dimension, which recorded 0.51, those of the other three dimensions ranged from 0.74 to 0.82. Generally, these reliability coefficients provide evidence of high precision of the teaching practice observation scores to a larger extent. This is particularly so because the coefficients obtained are consistent with recommendations from several authors (Shavelson and Webb, 1991; Brennan, 2001; Cardinet et al., 2010; Ho and Kane, 2013). Ho and Kane (2013), for example, recommend a minimum G-coefficient of 0.65 as sufficient for determining measurement precision, whereas others recommend 0.80 as an appropriate precision threshold (Shavelson and Webb, 1991; Cardinet et al., 2010). In a more specific sense, Brennan and Kane (1977) recommend that *Φ* ≥ 0.7 for research purposes, ≥0.8 for formative evaluations, and ≥0.9 for summative evaluations. Interestingly, the scores generated from the teaching practice exercise are used for both formative and summative purposes. Formatively, based on the scores and related feedback they receive, preservice teachers align their teaching with contemporary best practices. Regarding the summative purpose, scores are used to grade students, contributing to 80% of their semester GPA in the semester in which the teaching practice takes place. The findings of the current study are supported by the joint recommendation of the American Educational Research Association, American Psychological Association, and the National Council on Measurement in Education. They state that a higher degree of precision/reliability is warranted when a test score leads to a decision that is not readily reversible, such as the rejection or rejection of a candidate to a professional school (American Educational Research Association, American Psychological Association, National Council on Measurement in Education, 2014). Furthermore, George and Mallery (2003) suggest that the G- and dependability coefficients should be interpreted as Cronbach’s alpha in Classical Test Theory (CTT). Therefore, a coefficient of 0.70 is considered acceptable; 0.80 is considered good; and more than 0.90 is considered excellent. It is worth noting in this current study that the reliability estimates obtained from the G-theory analysis represent a general reliability estimate since errors emanating from items, occasions, and raters are simultaneously estimated; hence, the reliability coefficient is the net effect of these errors.

## Conclusion

We conclude that, overall, the regular off-campus teaching practice exercise for the 2020–2021 academic year measured preservice teachers’ teaching competencies with high precision. That notwithstanding, there were some errors associated with the process, some of which were accounted for, while others were not, due to how the teaching practice exercise was carried out. Furthermore, our study concludes that person-by-item interaction is a predominant source of measurement error and that the use of the same items for all preservice teachers during the teaching practice exercise could possibly blur the accuracy with which student teaching competencies were measured.

### Practical implications

Given the high generalizability coefficients found in this study, it is indicative that scores generated from the teaching practice exercise, to a great extent, could be interpreted soundly and meaningfully as depicting preservice teachers’ teaching abilities. As such, the summative use of those scores for grading and certification has significant implications for validity and reliability. Formatively, feedback from the teaching practice exercise could be used to improve these preservice teachers’ teaching abilities. Based on the optimizations from this study, it is imperative to suggest that the Centre for Teacher Professional Development of the university under investigation consider increasing the observation items to 28. They should also engage four supervisors, and supervision should be performed on five occasions to meet the acceptable level of dependability for summative evaluations (Brennan and Kane, 1977), as excessive observation may not significantly improve measurement precision but can only increase logistical costs. Additionally, this may also help address the perception of preservice teachers of the numerous supervisions as documented in the literature.

On the premise that person-by-item interaction contributed much error to the measurement. In practice, this may be due to the diverse needs of preservice teachers, coupled with their unique teaching abilities. The items in this case may not be doing well since all students are put in “one basket” and assessed with the same items. These items may not be responsive to the diverse needs of the preservice teachers. The center responsible for students’ teaching practicum is encouraged to possibly consider modifying and calibrating the observation protocol to suit students’ competency. This could be achieved with the use of IRT adaptive testing principles, where the items are calibrated based on the abilities of the preservice teachers, and the order of the items is contingent on the student’s performance on a previous item.

### Strengths and limitations

Our study is strengthened by the fact that only one study appears to have investigated errors in a teaching practice exercise in Ghana, and, more specifically, using G-theory. To the best of our knowledge, our study is among the first to account for multiple facets of errors in a teaching practice exercise at a university in Ghana. The optimization in this study informs policy on how to achieve optimal teaching practice outcomes with minimal logistics. We acknowledge that our study used data from a single academic year, which may limit generalization to other years. Additionally, teaching practice for practical lessons was not considered in this study; therefore, our findings should be interpreted with caution. Further, the study included only undergraduate students in the regular stream. Another limitation of the G-theory is that, since it is a group-level theory, we cannot probe the item difficulties for each person. Our study does not assume causality. Notwithstanding this, our study sets the pace and serves as a precursor to further investigations into the teaching practice exercise.

## Data Availability

The raw data supporting the conclusions of this article will be made available by the authors, without undue reservation.
